# CFD-Based Simulation Analysis for Motions through Multiphase Environments

**DOI:** 10.3390/biomimetics8060505

**Published:** 2023-10-23

**Authors:** Shuqi Wang, Jizhuang Fan, Yubin Liu

**Affiliations:** State Key Laboratory of Robotics and System, Harbin Institute of Technology, Harbin 150001, China; wangshuqi@hit.edu.cn (S.W.); fanjizhuang@hit.edu.cn (J.F.)

**Keywords:** CFD simulation, theoretical derivation, multiphase environments, fluid–solid coupling

## Abstract

The motion process and force of the jumper crossing a multiphase environment are of great significance to the research of small amphibious robots. Here, CFD (Computational Fluid Dynamics)-based simulation analysis for motions through multiphase environments (water–air multiphase) is successfully realized by UDF (user-defined function). The analytical model is first established to investigate the jumping response of the jumpers with respect to the jump angle, force, and water depth. The numerical model of the jumper and its surrounding fluid domain is conducted to obtain various dynamic parameters in the jumping process, such as jumping height and speed. Satisfactory agreements are obtained by comparing the error of repeated simulation results (5%). Meanwhile, the influence of the jumper’s own attributes, including mass and structural size, on the jumping performance is analyzed. The flow field information, such as wall shear and velocity when the jumper approaches and breaks through the water surface, is finally extracted, which lays a foundation for the structural design and dynamic underwater analysis of the amphibious robot.

## 1. Introduction

In recent years, an amphibious robot that can go through a multiphase medium environment has become a research hotspot [1,2]. It can simultaneously carry various sensors and equipment to move freely in a multiphase environment and has broad application prospects in environmental monitoring, underwater search and rescue, and other fields [3,4,5]. In the multiphase environment of water–air, amphibious robots can jump and move quickly to break through the surface of the medium by means of elastic rods or rotors [6,7]. However, the complexity and dynamics of the water–air environment pose great challenges to the design and control of robots. Therefore, it is of great significance to study the motion process of the robot across the multiphase environment and carry out hydrodynamic analysis to improve the application ability and control accuracy of the robot in complex environments.

At present, hydrodynamic analysis has been widely used in robot design and control [8,9,10]. Firstly, hydrodynamic factors need to be considered in structure design. When the robot moves in a water–air environment, it will be affected by water flow and water vapor resistance. Therefore, in the design process of the robot, the shape and material of the robot need to be considered to reduce the resistance [11]. In addition, the propulsion mode also needs to consider hydrodynamic factors to improve the mobility efficiency of the robot [12]. Secondly, the control of the robot needs to consider hydrodynamic factors [13]. It is necessary to adjust the moving speed and direction according to different water flow and water resistance to maintain a stable motion state. Thirdly, hydrodynamic analysis can help us evaluate the performance of the robot [14]. Through hydrodynamic analysis, the movement efficiency and stability of the robot in the water vapor environment can be evaluated, as well as the sensitivity of the robot to water flow and water vapor. This information can help us optimize the design and control system and improve the performance and adaptability of robots. In general, the existing hydrodynamic analysis of the robot is mostly concentrated in the single-phase medium motion. By analyzing the hydrodynamic characteristics of the underwater vehicle in the water, the researchers have understood the motion law and force in different flow velocities, flow directions and flow fields, and the influence of the robot on the environment. However, the cross-media motion characteristics and behavior of the robots are affected by many factors, such as the robot’s own attributes (structural parameters, shape, weight, etc.), waves, and flows on the water surface. Therefore, it is necessary to analyze the flow field characteristics of the jumping robot when it moves across the medium. 

According to the analysis of the existing literature, the hydrodynamic analysis of cross-media motion is mostly focused on the movement of objects into water [13,15,16,17]. The research ideas are based on the experimental results for theoretical modeling analysis to obtain the motion characteristics of a specific shape object; the change of the object shape will increase the complexity of the experiment, and it is impossible to further analyze the interaction and influence between the object and the fluid. In addition, due to the complexity and low efficiency of water experiments [18,19], the repeatability and universality of the experiments are greatly reduced, and the efficiency of simulation analysis can make up for this deficiency. It solves the problem that the experimental method cannot easily obtain the flow field information around the motion process [20,21,22,23]. The quantitative relationship between the robot’s own attributes, motion behavior, and performance can be quickly obtained by simulation analysis [24,25,26]. Therefore, the use of numerical simulation methods to test and analyze the hydrodynamic characteristics of the robot has gradually become one of the means commonly used by researchers [27,28,29]. As a fluid mechanics and multi-physics simulation software, Fluent has the advantages of high customization, high precision, high efficiency, and visualization, and can be used for simulation analysis of robot motion. By simulating the fluid environment around the robot, its motion characteristics and behavior in different fluid environments can be analyzed. Therefore, based on Fluent and using the UDF control function, the simulation process of the cross-media jumping motion of objects is successfully realized. The motion characteristics and flow field structure characteristics of the robot through the multiphase environment are discussed by simulation analysis, which provides reference and guidance for the design and control of the amphibious robot.

In the following part, an analytical model is first developed in theory to fully describe the physical process and force conditions of the cross-media motion. The motion simulation analysis method of robot crossing multiphase environment based on Fluent is introduced in Section 3, including the steps of establishing the robot model, establishing the fluid model, setting boundary conditions, carrying out simulation calculation, and analyzing simulation results. In the fourth part, the calculation results are verified, and the results are satisfactory. On this basis, the flow field structure characteristics of cross-media motion are obtained. Finally, a discussion of the results, as well as room for improvement, is provided.

## 2. Theoretical Analytical Modeling

**Figure 1:**
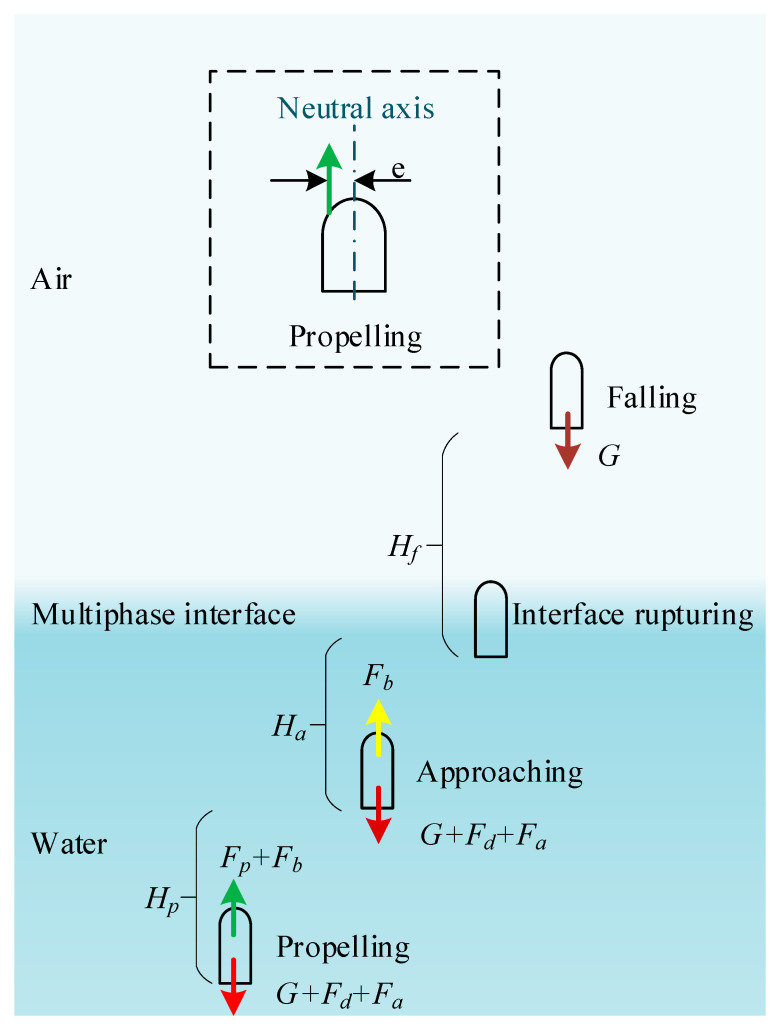
The analytical modeling principle of the jumpers: the propelling process, the approaching process, the interface rupturing process, and the falling deceleration process in air.

To analyze the force process of an object jumping through a multiphase environment theoretically, an analytical model that fully describes the vertical jumping process of an object is established. The shape of the object is simplified to a regular geometric shape, that is, a hemispherical shell head with a diameter range of 40–100 mm. The shell is cylindrical, ranging from 50 to 80 mm in length, and the diameter is the same as the shell head. The analytical modeling principle of the object is given in Figure 1, including the process of propelling, approaching, interface breaking, and rising to the highest point. In general, the jumper can achieve higher acceleration through rapid energy release during the propulsion process. The speed reached in the propulsion stage determines the displacement and speed of the entire motion cycle.

To provide a more concise analysis of the changes in the resultant force applied to an object during water movement, the formula symbols used in each stage are first simplified based on Figure 1, as shown in Table 1.

As the stored energy begins to release, the accelerating process of the object begins, which is rapid and short-lived (e.g., the time of the frog’s propulsion is about 0.1 s). When the reaction force generated by the released energy disappears, the object continues to move upward and gradually approaches the interface. The interface rupture process begins when the object head contacts the water surface, which is divided into the initial stage of the interaction between the object head and the multiphase interface and the end stage of the object completely leaving the water surface. After the boundary layer falls off, the object performs a uniform deceleration motion under its own gravity until the speed is zero and begins to land freely. Next, we will analyze the resultant force of each stage.

The force acting on the object during acceleration is as follows:

(1) $$F_{r}=F_{p}+F_{b}-F_{d}-F_{a}-G,t\in \left[0,\lambda \right]$$

where *F_r_*, *F_b_*, *F_p_*, *F_d_*, *F_a_*, and *G* are the resultant force of the jumpers, the buoyancy force, the thrust force, the drag force of water, the added mass force, and the gravitational force, respectively. λ is the applied time of the force. To make the robot hover in the water and reduce the influence of gravity, the size of buoyancy is generally designed to be equal to gravity as one of the basic design principles. Therefore, the influence of gravity and buoyancy will not be considered in the calculation of the resultant force before the interface ruptures.

The drag force *F_d_* is written as

(2) $$F_{d}=\frac{1}{2}\rho_{w}C_{d}SV^{2}$$

where *ρ_w_*, *C_d_*, *S*, and *V* represent the density of water, the drag coefficient, the upstream area, and the velocity of the jumpers. Based on a reference study about drag coefficient [30,31,32,33], *C_d_* is defined as a function of time as

(3) Cdt=ARet×1+φRet

where *A* and Re are the viscous drag coefficient and the Reynolds number, respectively. *φ* is the shape drag coefficient, which is related to the distance between the force and the neutral axis. It can be seen from Figure 1 that e is the eccentricity of the object, which takes zero when the force passes through the center line of the object.

The Re is a certain characteristic dimensionless quantity related to the velocity, density, and viscosity coefficient of the fluid, which can be written as

(4) Ret=ρwL⋅Vtμ

where *L* and *µ* are the characteristic length of the jumpers, regarded as the diameter of the object, and the dynamic viscosity of water that equals 1.01 × 10^3^ Pa·N, respectively. When the Reynolds number is less than the critical value (usually 2000), the fluid is in a laminar flow state with strong viscosity and relatively stable fluidity; when the Reynolds number is greater than the critical value, the fluid is turbulent. Due to the relatively high speed that needs to be generated during the propulsion process to break through the water surface, the time-averaged Reynolds number is larger than 3 × 10^4^, which is high enough such that the viscous drag term is omittable in Equation (5). Additionally, the added mass force can be calculated as

(5) $$F_{a}=\alpha \rho_{w}J_{v}\frac{dV}{dt}$$

where *α* is the added mass coefficient, and *J_v_* is the volume of the jumpers. It is noteworthy that the added mass coefficient also depends on the shape factor of the jumpers.

The approaching process of the jumpers can be written as

(6) $$F_{r}=-F_{a}-F_{d},t\in \left(\lambda ,t_{1}\right)$$

where *t*_1_ is the time that the jumper head touches the water surface.

Taking into account the average impact velocity and the average Weber number when rushing out of the water, the effect of surface tension on the object is negligible. At this time, the balance between gravity and buoyancy began to be broken. Thus, the preliminary stage of the jumping-out process can be written as

(7) $$F_{r}=-F_{a}-G+\rho_{w}gh_{in}J_{vin}$$

where *h_in_* and *J_vin_* are the underwater height and the volume of the jumpers, respectively.

Finally, the object continues to move upward after leaving the water surface. The speed of the object gradually decreases to zero under the action of its own gravity, and then the falling process begins.

**Figure 2:**
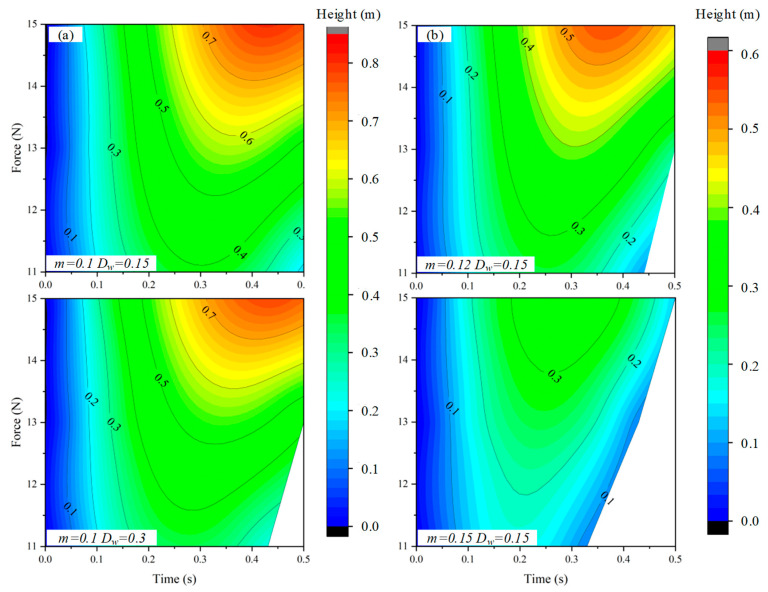
The theoretical relationship analysis between the various factors. (**a**) The relation between thrust force, water depth, and displacement. (**b**) The influence of mass and thrust force on displacement.

According to the theoretical analysis, the influence of force, mass, and water depth on the jump height is obtained, as shown in Figure 2. There is no doubt that the jumping height of the object increases with the increase of the driving force. In the case of a certain thrust force provided by the driving mechanism, the influence of motion parameters (water depth) and structural parameters (mass) on the motion performance of the object is analyzed. The critical value of the blank part in the figure is water depth, which means that the jumper has fallen from the highest point to the water surface. In the case of constant mass and double water depth, the area of the blank part is about 0.05 of the overall area. However, the area of the blank part is increased by four times when the water depth is increased by 0.05 kg. It can be intuitively seen that the influence of mass on the jump height is much greater than the water depth, especially from the comparison of the blank part of Figure 2b. This is because the weight leads to a very low jump height, which falls into the water very early. Therefore, only considering the jump height of the object, the internal factors of the object should be considered compared with the external factors. The following will verify the correctness of the theoretical analysis model by simulation analysis. On this basis, the influence of these factors on the robot crossing the medium will be further analyzed, and reference suggestions for improving the motion performance of the robot will be provided according to the simulation results.

## 3. Numerical Modeling

### 3.1. Subsection Modeling and Mesh

**Figure 3:**
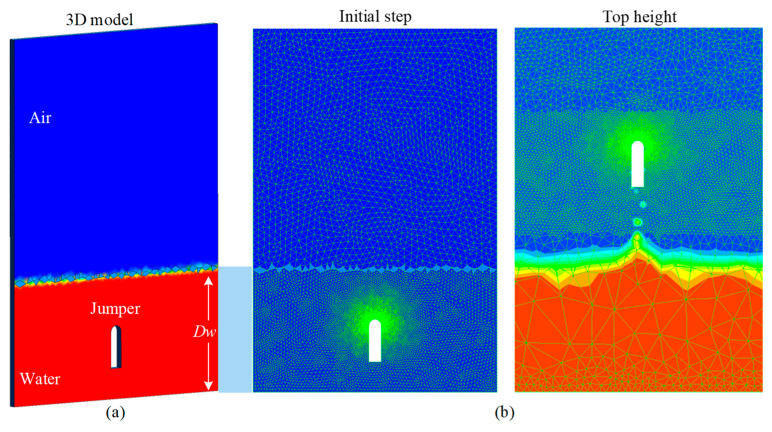
The numerical modeling principle of the jumpers. (**a**) Multiphase model in initialization state. (**b**) Mesh subdivision at the initial time and the highest point.

The jumping process of the soft jumpers is numerically simulated in FLUENT, ANSYS. For the hydrodynamic simulation, a 3D numerical model is developed, as shown in Figure 3a. Pre-processing to mesh the fluid field and jumper surface was needed before calculations were conducted. The volume mesh and the boundary surface mesh of the calculation area in the initial state are shown in Figure 3b. The volume of phases model is used to solve the multiphase fluid dynamics phenomenon. The lower fluid is set to viscous incompressible water, and the initial flow field velocity is zero.

The CFD calculation space was bounded by the flow field boundary and the inner boundary formed by the jumper model. Due to the high impact velocity when the jumper jumps out of the water surface, the surrounding flow field is seriously disturbed. Therefore, the dynamic mesh method is utilized to control the jumper grid movement in the numerical simulation, which allows the elements to deform using the spring performance assumption. When the element deformation reaches the threshold, the global mesh is automatically re-mesh to ensure the accuracy of the calculation.

### 3.2. Mesh Simulation Setup and Method

The simulation conditions are set after the meshing is completed. It should be noted that during the movement, since the moving object is only a relatively small object in the system, it can be set based on the Euler method so that the fluid domain (grid) inside is moving, and the top and bottom are fixed, respectively. To overcome the reaction forces such as drag force, additional mass force, and gravity during the jump process, it is necessary to apply a transient force (i.e., the thrust force generated by energy release) at the bottom of the model to ensure that the object can successfully jump out of the water. It was operated in the FLUENT macro named DEFINE_CG_MOTION. 

The governing equations are the time-averaged incompressible continuity equation and the Navier–Stokes (N–S) equation. The standard k−ε viscous model is used to ensure the calculation stability. According to the minimum grid size near the top of the model, the simulation step size is set to 0.0001 s so as to ensure the small displacement of the grid, which is helpful to realize the dynamic grid and the convergence of the calculation. The semi-implicit method for the pressure-linked equation algorithm is used to solve the coupling problem between the pressure and velocity. And detailed information about the numerical simulations is shown in Table 2.

### 3.3. Smulation Process

**Figure 4:**
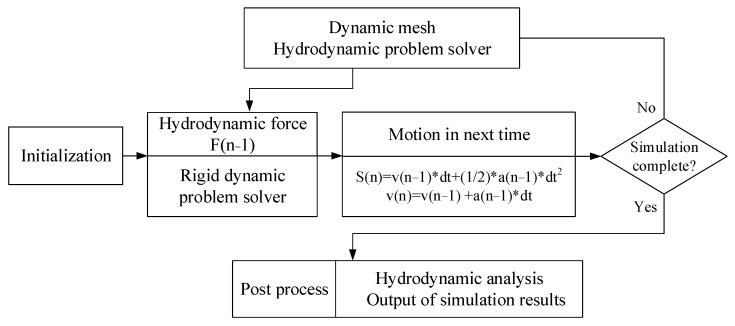
The simulation process of jumping through a multiphase environment.

The FLUENT solver calculates the hydrodynamic problems in the simulation of a multiphase environment. Taking the jumper model as the inner boundary, the dynamic mesh technology is used to control the movement of the model. These steps can be easily carried out in FLUENT. Users can integrate grid control, dynamic computing, data processing, etc., in the UDF. The detailed simulation process is shown in Figure 4.

Firstly, the fluid and boundary conditions are initialized. Then, governing equations are used to calculate the flow field information and solve the hydrodynamic problem. UDF receives the hydrodynamic results and uses them to calculate the rigid dynamics of the object. By calculating the dynamic problem, the motion of the object at the next moment can be derived. Finally, the input data used to reset the boundary conditions and control the dynamic mesh are refreshed to start the next calculation cycle. After repeated calculations, the fluid–solid coupling problem is solved. The kinematics and dynamics result of the object and the flow field information are obtained.

## 4. Result

### 4.1. Simulation Method Estimation

**Figure 5:**
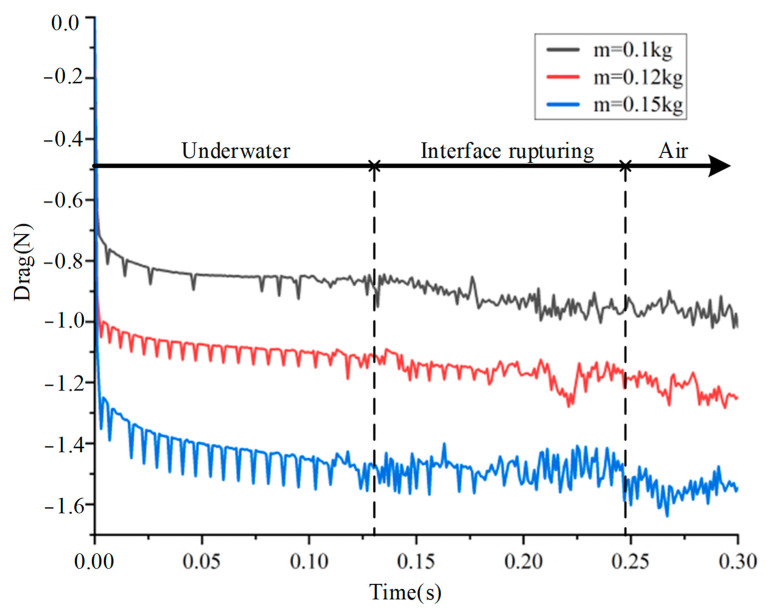
The resistance changes of objects with different masses in the process of simulation motion.

As an important influencing factor in the analysis of cross-media locomotion, the resistance is first extracted to evaluate the correctness of the simulation method and results. Since the theoretical resistance in the air is only its own gravity, the structural parameters and motion parameters are controlled unchanged, and the resistance changes with different masses during the movement are extracted, as shown in Figure 5.

It can be seen that the force curve is basically the same in the development trend, showing a three-stage type, including underwater, interface rupturing, and air. At the beginning of the locomotion, the resistance begins to increase rapidly and then remains stable until it contacts the water surface. At about 0.13 s, it began to break through the water surface, and the balance between buoyancy and gravity was broken. The decrease of buoyancy and viscous force led to the gradual increase of resistance. After completely leaving the water, the resistance fluctuates around the gravity and tends to be stable. In addition, a small amount of water attached to the object will cause the resistance to increase slightly, which is in line with the actual situation.

Although there are discrepancies in the force curves, the simulation method is still successful in calculating fluid dynamics problems and analyzing the locomotion process. According to the current research results, the calculation results meet the requirements and can be improved by better adjusting the initial conditions or grid quality.

### 4.2. Structure Parameter Analysis

**Figure 6:**
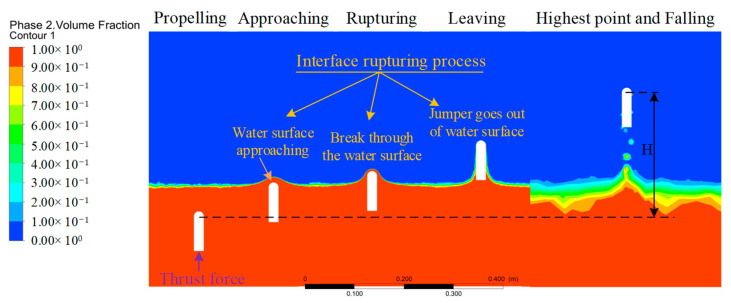
The simulation process of vertical crossing multiple environments at different times.

The factors that affect the motion performance of objects are divided into internal factors (structure parameter) and external factors (motion parameter). The hydrodynamic force of the jumper has a great influence on its motion when it moves amphibiously, especially when it crosses multiple environments. Therefore, it is very important to explore appropriate structural parameters to reduce water resistance and improve motion performance from the perspective of internal factors. Here, the influence of the internal factors of the jumper on the water jump performance was first analyzed. To obtain the relationship between the jump height and the mass, diameter, and overall length of the object, a series of water jump simulations were performed. The vertical motion process through the multiphase interface is shown in Figure 6. The object begins to move under the action of an instantaneous propulsion force, including the process of accelerated propulsion, interface breakthrough, air flight, and landing, in which the interface breakthrough includes the water surface approaching, breaking through the water surface, and going out of the water surface.

The mass range of the object is 0.1–0.15 kg, the radius is 20–50 mm, and the length range is 50–80 mm. Note that each simulation is performed under the same constraints as the external conditions, and repeated verification is performed. The maximum height is defined as the distance difference between the jumper’s head from the initial position to the beginning of the landing. The verification results show that the maximum jump difference of a set of simulations is less than 5%, indicating the consistency of the simulation results and that its subtle influence can be ignored.

**Figure 7:**
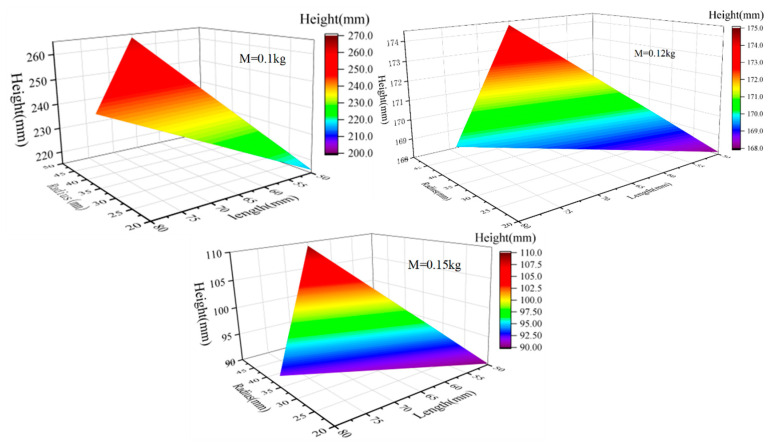
The influence of structural parameters, including mass, radius, and length, on motion performance.

The relationship density diagram of structural parameters obtained by multiple sets of simulations is shown in Figure 7. Generally, the object can jump 0.17 m on average and can jump 0.26 m at most. When the object is set to a certain width and length, the jump height increases with the decrease in mass, which can be directly seen from the amplitude of each figure. Consistent with the theoretical analysis, the mass has the greatest influence on the jumping performance of the object. Therefore, selecting the appropriate materials to realize the miniaturization and lightweight of the robot needs to be considered first in the structural design process. On the other hand, the jump height increases faster as the radius increases, which indicates that the radius of the object plays a leading role compared with length in the multiphase jump locomotion when its own mass is certain. This is because a large radius can quickly change the balance between gravity and buoyancy and reduce water resistance after breaking through the interaction surface of the medium when the volume is constant.

### 4.3. Motion Parameter Analysis

**Figure 8:**
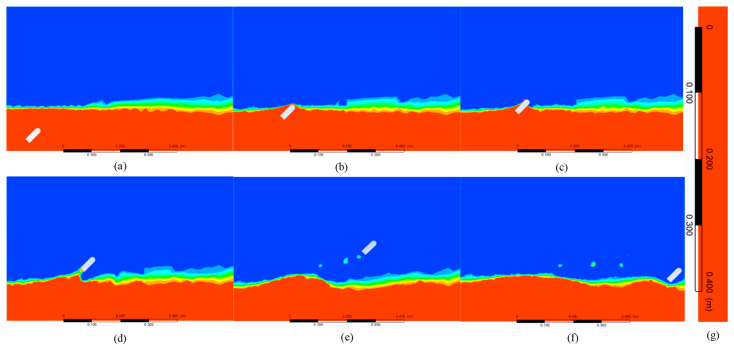
Simulation results of the object jumping out of the water at a motion angle of 45°, including accelerated propulsion (**a**), interface breakthrough (**b**,**c**), air flight (**d**,**e**), and landing (**f**). Local enlarged diagram of coordinate system (**g**).

As an external factor, the influence of motion parameters on jumping height cannot be ignored. Therefore, the influence of the motion parameters of the jumper on the motion performance is further analyzed after determining the structural parameters. To better reflect the jumping performance, the jumper’s own properties are set to the best motion performance when analyzing external factors. The case where the object jumps out of the water at a motion angle of 45° is shown in Figure 8. Similar to the process of vertical movement of the object, it has also experienced accelerated propulsion, interface breakthrough, air flight, and landing. The jumping movement of the inclined angle can make the jumper produce a certain displacement in the horizontal direction and then realize the obstacle avoidance movement, such as crossing the obstacle, which is more practical than vertical take-off. Therefore, it is necessary to explore the influence of motion parameters on jumping performance and then extract the flow field information in the process of motion for hydrodynamic analysis on this basis.

**Figure 9:**
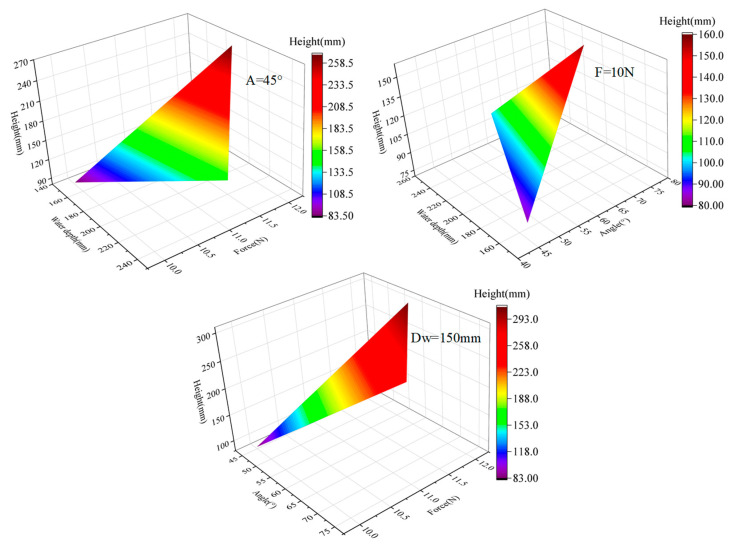
The influence of motion parameters, including jumping angle, force, and water depth, on motion performance.

The simulation of multiple jumps is carried out, and the cloud diagram of the influence of force, water depth, and jump angle on the jump height can be obtained, as shown in Figure 9. The range of force is 10–12 N, the range of water depth is 150–250 mm, and the range of jump angle is 45–75°. It should be noted that this is the angle between the direction of motion of the jumper and the horizontal line. Through the longitudinal comparison with the amplitude when analyzing the structural parameters, it can be intuitively seen that under the premise of selecting the optimal structural parameters, the motion parameters have greatly improved the jumping performance of the object, and the highest jumping height has reached 0.34 m, which also shows the importance of optimizing the motion parameters from the side. Then, the influence of the interaction between the motion parameters on the motion performance is compared horizontally. It can be seen that the amplitude of the jump height is about half of the other two parameters when the value of the force is constant, which indicates that the force has the most direct influence on the motion performance, which is also consistent with the results in the theoretical analysis. However, there are four extreme special situations to explain first. When the water depth is 0 m (i.e., single phase of the air) or the jumping angle is 90° (i.e., vertical motion), the jumping height is the highest, and when the depth tends to infinity (i.e., single phase of the water) or the angle is 0° (i.e., parallel to the water surface) the jumping height is the lowest. Therefore, it is impossible to intuitively judge which one has a greater impact on the performance of the movement.

The orthogonal experimental analysis is further carried out to study the dominant role of motion angle and water depth in multiphase motion. Range analysis can be used to study orthogonal test data, including the advantages of factors or their specific levels. The results of the range analysis are shown in Table 3. Among them, *K* is the sum of test data at a certain level of a certain factor. *K_a_* is the corresponding average value. *R_f_* is the range of factors.

**Figure 10:**
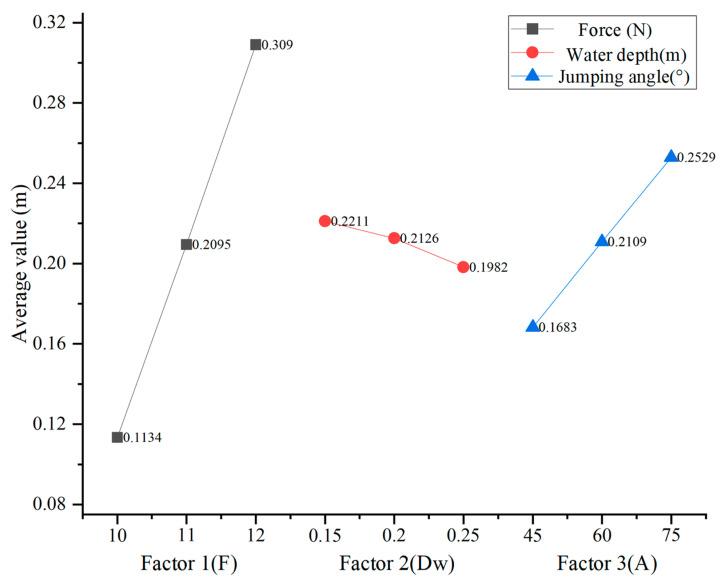
The average value of each level of factor.

As shown by the theoretical analysis and the actual situation, the jump height of the object increases with the increase of the force, which indirectly verifies the correctness of the simulation results. By comparing the maximum jump under different water depths and jump angles, the maximum jump law with the increase of water depth and angle is obtained. To view the influence of each level of the factor intuitively, the average value of each factor is shown in Figure 10. From the perspective of three factors, combined with the comparison of R value (factor range), factor 1 force is the optimal factor, followed by factor 3 jump angles, and finally, factor 2 water depth. Therefore, the order of the three factors was factor 1 (force) > factor 3 (jump angle) > factor 2 (water depth). According to the optimal level of each factor, the third level of factor 1 is 12 N, the water depth of factor 2 is 0.15 m, and the jump angle of factor 3 is 75°.

The results of orthogonal experiment analysis show that compared with water depth, jump angles play a leading role in the multiphase jump motion of the object under the condition of a certain force. Therefore, the driving unit with high energy density should be selected first in the design of the robot driving mode. Without considering the jump distance, the jump angle is selected as large as possible by the attitude adjustment structure, and the suspension motion is gradually close to the water surface before starting to jump so that the best motion effect will be obtained.

### 4.4. Hydrodynamic Analysis

**Figure 11:**
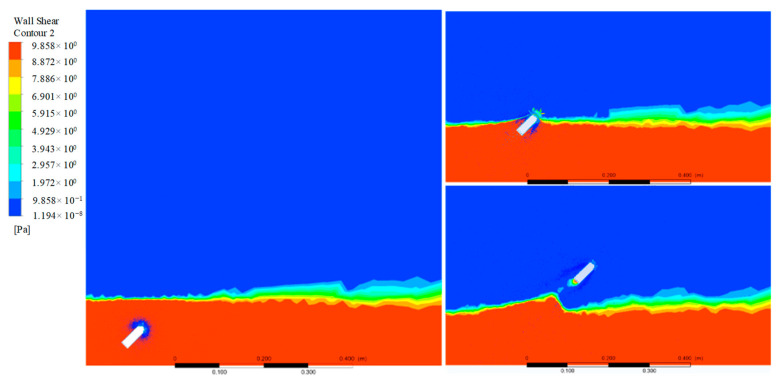
The extracted contour of wall shear of the jumper when the jumping angle is 45°.

The flow field information of the object in the motion process will be extracted for the following fluid analysis by taking the jumping angle of 45° as an example. The force area of the jumper has a great influence on the water resistance in the process of water movement. Therefore, the contour of the wall shear is extracted and analyzed first, as shown in Figure 11. It can be seen that the shear force is mainly concentrated in the head of the arc at the beginning of the locomotion. When it is close to the water surface, the force balance on both sides of the jumper is broken due to the problem of the movement angle, and the force below gradually increases to produce a clockwise torque, which further reduces the movement angle. After the object jumps out of the water, it is mainly concentrated in the tail. It can also be seen from the whole movement process that the shear stress of the object is mainly concentrated at both ends, and the two sides of the object are smaller, indicating that the length of the object has little effect on the shear stress. For its structural parameters, the diameter has a greater performance on the object than the length.

**Figure 12:**
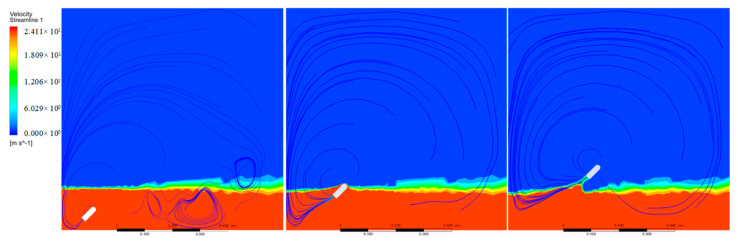
The extracted velocity field information of the jumper at different motion stages.

The velocity of movement is the result of the combined action of multiple forces. The streamline of the velocity field of the object passing through the water surface is shown in Figure 12. It can be seen that at the beginning of the movement, the streamline of the distal water surface is denser, and the water surface is constantly lifted, which is equivalent to increasing the initial water depth and affecting the jumping height of the object. As the object continues to rise, the streamline at the tail gradually becomes dense. The tension and inertia of the medium make it continue to follow the object for a certain distance, that is, the water column below the object, which also has a certain impact on the jumping performance of the object. From the streamline of the velocity field, the jumping angle indirectly changes the water depth to affect the jump height of the object. Therefore, the angle has a greater impact on the motion performance of the object than the water depth.

## 5. Discussion and Conclusions

The CFD-based simulation of the movement process of the jumper through multiple environments was realized in this study combined with UDF. The whole jumping process, including the interface rupturing process and the influence of structural parameters and motion parameters on the jumping performance, is analyzed theoretically and numerically. Theoretical analysis shows that the mass of the jumper and the force are the main factors affecting the motion performance, so we can first consider these two aspects to improve the motion ability. The numerical simulation is carried out on this basis, and the repeated results are less than 5%, which verifies the rationality of the simulation method. Meanwhile, the velocity of the object decreases rapidly during the process of interface rupture due to the decrease of buoyancy, which is consistent with the actual physical phenomenon [34]. The simulation results show that the diameter of the jumper has a greater influence on the motion than the length in the structural parameters. The influence of motion parameters on the jump height is further analyzed using the orthogonal experiment method. Compared with water depth, reducing the jumping angle is more helpful in improving the motion performance. The flow field structure information in the process of the jumper breaking through the interface is finally extracted, including the stress field and velocity field. The interaction between the object and the air–water interface has been analyzed for its jumping performance, which provides a theoretical basis and reference for the subsequent selection of structural and motion parameters.

**Figure 13:**
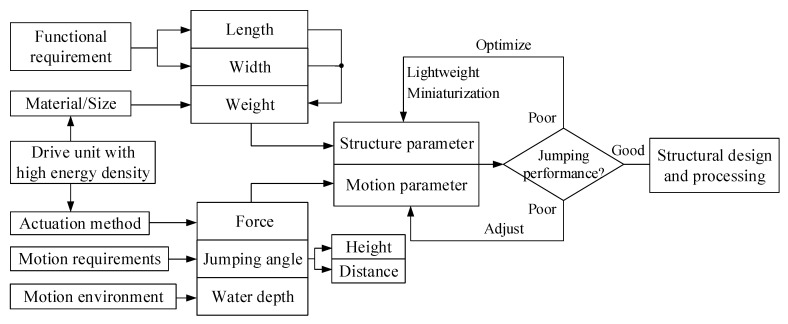
Analysis of influencing factors of jumping performance and countermeasures.

Based on the simulation results, the influencing factors of jumping performance are summarized and analyzed, and the corresponding countermeasures are put forward, as shown in Figure 13. Among them, the weight of the object and its thrust force have the greatest influence on the motion performance and are also the primary factors to be considered in the structural design. The following four suggestions are provided for the design of cross-media robots for reference: The new material lays a foundation for optimizing and improving the structural design of the amphibious robot. It is necessary to consider the combination of new materials to realize the lightweight of the robot in the structural design. Secondly, the robot should be as close to the water surface as possible to further improve the motion performance when its task is not considered. Then, the jump angle of the robot should be adjusted according to the height of the obstacle to meet different locomotion requirements. Finally, we can see that the driving unit with high energy density not only affects the driving mode and then affects the value of the propulsion force, but it also has a direct impact on the weight of the object. This unit is the key to realizing miniaturization and explosive motion and will be one of the key contents of the research on underwater jumping robots. 

It is worth noting that the influence of the structural parameters of the object on its motion performance was obtained through simulation analysis, providing a macro perspective for structural design. However, there are certain differences between the structure used in the simulation process and the actual structure of the jumping robot, and the final structural parameters cannot be determined in detail. Therefore, conducting structural design on this basis, obtaining the stress concentration area during the motion process through simulation analysis, and then conducting feedback optimization of the structure will be a good research approach. Secondly, the simulation results can also improve accuracy on the existing basis, such as improving the quality of the grid and shortening the time step, but this will increase the simulation time and affect efficiency. In addition, we only conducted parameter analysis from the perspective of simulation, and it is also necessary to obtain data through testing experiments to further optimize simulation parameters and verify the correctness of the simulation.

## Figures and Tables

**Table 1:** Nomenclature of symbols.

Symbol	Implication	Symbol	Implication
*F_r_*	resultant force	*F_d_*	drag force
*F_p_*	thrust force	λ	applied time of the force
*F_b_*	buoyancy force	*ρ_w_*	density of water
*F_a_*	added mass force	*C_d_*	drag coefficient
*G*	gravitational force	*S*	upstream area
*V*	velocity	*α*	added mass coefficient
*A*	viscous drag coefficient	*J_v_*	volume of the jumper
*Re*	Reynolds number	*h_in_*	underwater height
*φ*	shape drag coefficient	*J_vin_*	underwater volume

**Table 2:** Detailed information in the numerical simulations.

Mesh	Node	6237
	Elements	12,024
	Size function	Proximity and curvature
	Minimum size (mm)	1.0008
	Maximum face size (mm)	2.6903
Multiphase model	Model	Volume of fluid
	Number of Eulerian phases	2
	Volume fraction parameters	Implicit
	Volume fraction cutoff	1 × 10^−6^
Viscous model	Model	Standard k−ε
	*C_µ_*	0.090
	*C*_1*ε*_	1.440
	*C*_2*ε*_	1.920
	*σ_k_*	1
	*σ_ε_*	1.300
Solution methods	Scheme	PISO
	Neighbor correction	1
	Skewness correction	1
Parameters for jumpers	M (kg)	0.1/0.12/0.15
	R (mm)	20/35/50
	L (mm)	50/65/80
Parameters for fluid	*σ*	0.072
	*D_w_* (mm)	150/200/300

**Table 3:** The results of range analysis.

Items	Level	Factor 1 (F)	Factor 2 (D_W_)	Factor 3 (A)
K	1	0.3402	0.6632	0.5048
	2	0.6285	0.6379	0.6324
	3	0.9271	0.5947	0.7586
K_a_	1	0.1134	0.2211	0.1683
	2	0.2095	0.2126	0.2109
	3	0.3090	0.1982	0.2529
Best level		3	1	3
R_f_		0.1956	0.0228	0.0846
Number of levels		3	3	3
Number of level repetitions r		3	3	3

## Data Availability

The datasets generated during simulation or analyzed during the current study can be obtained from the corresponding author according to reasonable requirements.
